# Development of subfamily-based consensus PCR assays for the detection of human and animal herpesviruses

**DOI:** 10.1007/s10096-023-04605-w

**Published:** 2023-04-21

**Authors:** God’spower Richard Okoh, Michelle Lockhart, Joanne Grimsey, David Whitmore, Ellen Ariel, Jeff Butler, Paul F. Horwood

**Affiliations:** 1 College of Public Health, Medical and Veterinary Sciences, James Cook University, Townsville, Queensland 4811 Australia; 2 CSIRO Australian Centre for Disease Preparedness, Geelong, Victoria 3220 Australia

**Keywords:** Herpesvirus, Consensus PCR, DNA polymerase gene, Glycoprotein B gene

## Abstract

Consensus PCR assays that can be used to sensitively detect several herpesvirus (HV) species across the different subfamilies were developed in this study. Primers containing degenerate bases were designed to amplify regions of the DNA polymerase (DPOL) gene of alpha- and gamma-HVs, and the glycoprotein B (gB) gene of beta-HVs in a singleplex, non-nested touchdown PCR format. The singleplex touchdown consensus PCR (STC-PCR) was used to amplify the DNA of eight human and 24 animal HVs. The assay was able to detect the lowest DNA dilution of 10^−5^ for alpha-HVs and 10^−3^ for beta- and gamma-HVs. In comparison, lowest detection limits of 10^−5^, 10^−3^, and 10^−2^ were obtained for alpha-, beta-, and gamma-HVs respectively when a nested PCR was used. The findings in this study suggest that the STC-PCR assays can be employed for the molecular surveys and clinical detection of novel and known HVs.

**Supplementary Information:**

The online version contains supplementary material available at 10.1007/s10096-023-04605-w.

## Introduction

Herpesviruses (HVs) are known to have a wide host range, infecting both vertebrate and invertebrate species [1, 2]. The virus is made up of a linear, monopartite, double-stranded DNA genome that encodes up to 300 genes and ranges from 124 to 241 kbp in length [3]. Herpesviruses are divided into three subfamilies, the *Alpha*-, *Beta*-, and *Gamma*-*herpesvirinae* on the basis of biological and molecular properties [2]. A common feature among all of the sub-groups of HVs is their ability to cause latent infection in infected hosts, which can be reactivated to cause serious illness in immunocompromised hosts [2]. Clinical diseases associated with active or recrudescent HV infections vary according to the hosts and the infecting viral species. For instance, the human HVs (HHV-1 to HHV-8) are members of *Alpha*-, *Beta*- and *Gamma*-*herpesvirinae* and have been associated with gingivostomatitis, herpetic keratitis, encephalitis, varicella, mononucleosis, lymphoproliferative malignancy, roseola and sarcoma [2]. The HVs of ruminants belong to the subfamilies *Alpha*- and *Gamma*-*herpesvirinae*, and infections are associated with rhinotracheitis (ovine HV1, caprine HV1), herpes mammalitis (bovine HV2), meningoencephalitis (bovine HV5), fatal systemic infection (caprine HV1), malignant catarrhal fever (ovine HV2, alcelaphine HV1, 2), ocular disease (cervine HV1) and fatal neurological disorder (bubaline HV1) [4]. The avian and reptilian HVs have so far only been assigned to the subfamily *Alphaherpesvirinae* causing clinical and economic important diseases such as Marek’s disease (gallid HV2) and infectious laryngotracheitis (gallid HV1) in poultry, duck plaque enteritis (anatid HV1) in waterfowl, Pacheco’s disease (psittacid HV1) in psittacines and fibropapillomatosis (chelonid HV5) in sea turtles [5–10]. Mixed infections of HV species can occur in susceptible hosts leading to a variety of clinical symptoms that may be difficult to diagnose or treat [11–15]. Therefore, there is a need for a sensitive assay that can reliably detect HV species of more than one subfamily in the same clinical samples.

Molecular surveys often employ consensus PCR assays to detect known and novel HVs [16–19]. In fact, several new HV species of mammals, reptiles and avians have been discovered using this approach [18, 20–25]. Despite these valuable outcomes, some of the existing consensus PCR assays have variable sensitivity to different HV subfamilies and require a nested PCR format, which can be costly and prone to contamination. Here, we have designed singleplex touchdown consensus PCRs (STC-PCRs) that amplify regions of the DNA polymerase (DPOL) gene of alpha- and gamma-HVs and glycoprotein B (gB) gene of beta-HVs. This non-nested PCR assay was successfully used to detect a wide range of HVs across a broad range of herpesviruses in two independent laboratories.

## Materials and methods

### Primer design

Degenerate consensus primers were designed for each subfamily based on the alignment of full and partial nucleotide sequences of HVs obtained from GenBank (Supplementary file 1, Table S1). The HV sequences were imported into Geneious 11.1.5 (https://www.geneious.com), and alignments were conducted with ClustalW 2.1 using the default parameters. Primers (Table 1) were manually generated from the conserved regions following visual inspections of the alignments.

**Table 1:** List of consensus herpesvirus primers designed for this study

Subfamily	Primer	Sequence (5′ → 3′)	Orientation	Gene	Product length (bp)
*Alphaherpesvirinae*	AlphaFWD1	AGCATHATYCAGGCBCAYAAYCTSTGYTTYA	Sense	DPOL	265-277
	AlphaREV2	TTRATBGCVRVCTGYTGYTTRTC	Antisense		
*Betaherpesvirinae*	BetaFWD_gb1	GARGCBTGGTGTHWVGATCA	Sense	gB	564
	BetaREV_gb1	YT[+C]YARR[+T]CRAANACGTT	Antisense		
*Gammaherpesvirinae*	GammaFWD1	GGVTAYAACRTNKSMAAYTTTGA	Sense	DPOL	650
	GammaREV1	GGRTASAGGCTRGCAAARTC	Antisense		

### DNA preparation and Singleplex Touchdown Consensus PCR

The human and animal HV DNAs tested in this study are shown in Table 2. Viral DNAs were extracted from infected tissues or culture supernatants using the DNeasy Blood and Tissue Kit (Qiagen) as recommended by the manufacturer. Additional DNA extracts were obtained from the Victorian Infectious Diseases Reference Laboratory (VIDRL).

**Table 2:** Human and animal herpesviruses tested by STC-PCR and the limit of detection of representative viral species

Subfamily (Genus)	Virus	Results (limit of detection*)		Sample type
		STC-PCR	Nested-PCR [19]	
*Alphaherpesvirinae*				
Simplexvirus	*Human alphaherpesvirus 1*^a^	+ (10^−3^)	+ (10^−3^)	Human clinical sample
	*Human alphaherpesvirus 2*^a^	+	+	Human clinical sample
	*Macropodid alphaherpesvirus 1*^a^	+	+	Cell culture isolate
	*Macropodid alphaherpesvirus 2*^a^	+	+	Cell culture isolate
	*Bovine alphaherpesvirus 2*^b^	+	+	Cell culture isolate
Iltovirus	*Gallid alphaherpesvirus 1*^b^	+	+	Cell culture isolate
Mardivirus	*Columbid alphaherpesvirus 1*^a,b^	+ (10^−4^)	+ (10^−4^)	Animal clinical sample; Cell culture isolate
	*Meleagrid alphaherpesvirus 1*^a,b^	+	+	Animal clinical sample; Cell culture isolate
	*Gallid alphaherpesvirus 2*^a,b^	+	+	Animal clinical sample; Cell culture isolate
	*Anatid alphaherpesvirus 1*^b^	+	+	Cell culture isolate
Varicellovirus	*Bovine alphaherpesvirus 1*^a,b^	+ (10^−5^)	+ (10^−5^)	Cell culture isolate
	*Human alphaherpesvirus 3*^a^	+	+	Human clinical sample
	*Equid alphaherpesvirus 1*^b^	+	+	Cell culture isolate
	*Equid alphaherpesvirus 3*^b^	+	+	Cell culture isolate
	*Equid alphaherpesvirus 1*^b^	+	+	Cell culture isolate
	*Felid alphaherpesvirus 1*^a,b^	+	+	Cell culture isolate; Vaccine (F3)
Scutavirus	*Chelonid alphaherpesvirus 5*^a^	+	+	Animal clinical sample
Unassigned/unknown	Crocodyline herpesvirus 1^a^	+ (10^−5^)	+ (10^−5^)	Cell culture isolate
	Crocodyline herpesvirus 2^a^	+	+	Cell culture isolate
	Crocodyline herpesvirus 3^a^	+	+	Cell culture isolate
	Phascolarctid herpesvirus^a^	+	+	Animal clinical sample
	Avian herpesvirus	+	+	Cell culture isolate
*Betaherpesvirinae*				
Cytomegalovirus	*Human betaherpesvirus 5*^a^	+ (10^−3^)	+ (10^−2^)	Human clinical sample
Roseolovirus	*Human betaherpesvirus 6*^a^	+ (10^−2^)	+ (10^−3^)	Human clinical sample
	*Human betaherpesvirus 7*^a^	+	+	Human clinical sample
Gammaherpesvirinae				
Lymphocryptovirus	*Human gammaherpesvirus 4*^a^	+ (10^−3^)	+ (10^−1^)	Human clinical sample
Manticavirus	*Phascolarctid gammaherpesvirus 1*^a^	+ (10^−3^)	+ (10^−2^)	Animal clinical sample
Rhadinovirus	*Human gammaherpesvirus 8*^a^	+	+	Human clinical sample
	*Bovine gammaherpesvirus 4*^b^	+	+	Cell culture isolate
Percavirus	*Equid gammaherpesvirus 2*^b^	+	+	Cell culture isolate
Macavirus	*Ovine gammaherpesvirus 2*^b^	+	+	Cell culture isolate

*The limit of the detection was recorded for the representative HVs tested

^a^Viruses tested at James Cook University (JCU) laboratory

^b^Viruses tested at The Australian Centre for Disease Preparedness (AAHL) laboratory

Following assay optimisation (data not shown), the STC-PCR was used to amplify HV DNA in a 20-μL reaction. The reaction mix contained 2 μL of DNA template, 1 μM (beta-HV) or 2 μM (alpha-HV and gamma-HV) primers (Table 1), 200 μM of each deoxynucleotide triphosphate (dNTP), 1.5 mM MgCl_2_, 0.5 U of HotStarTaq polymerase and 1× PCR buffer (Qiagen). The assays were successfully evaluated with ready-to-use pre-mixes including the GoTaq Hot Start Green Master Mix (Promega) and the HotStarTaq Plus master mix (Qiagen) to ensure the assays could be used across a range of PCR chemistries (data not shown). PCR enhancers, including 5% dimethyl sulfoxide (DMSO) and tetramethylammonium chloride (15 mM; TMAC), were also added to the reaction mix. A Touchdown PCR protocol was carried out as outlined in Table 3. The PCR products were analysed on a 1.5% agarose gel made up of 1× TBE buffer and 1× GelRed nucleic acid stain (Biotium).

**Table 3:** Optimised touchdown cycling protocol

	Temperature (°C)	Duration	Comments
1. Initial denaturation	95	15 min	Denaturation time vary with master mixes (according to the manufacturers’ instruction)
15 cycles of :			
2. Denaturation	94	1 min	
3. Annealing	63 decrement by 1 °C per cycle (− 1 °C/cycle)	1 min	
4. Extension	72	2 min	
35 cycles of:			
5. Denaturation	94	1 min	
6. Annealing	48	1 min	
7. Extension	72	2 min	
1 cycle of:			
8. Final extension	72	10 min	
1 cycle of:			
9. Hold	4	Infinity	

The specificity of the herpesvirus consensus assays was evaluated by testing a large number of alphaherpesviruses (*n* = 22), betaherpesviruses (*n* = 3) and gammaherpesviruses (*n* = 6). The assay performance was compared to another commonly used herpesvirus nested consensus PCR [19]. The STC-PCR relative sensitivity was tested by assaying a series of 10-fold dilutions of the DNA extracts of representative HVs from each subfamily, and comparing the limit of detection (LOD) with the VanDevanter assay [19]. The assay specificity was also checked by testing the consensus primer pair of one subfamily with the HV DNA templates of other subfamilies. To assess the reproducibility of the assay, herpesviruses were tested using the assays at two independent laboratories, with 22 viruses tested at James Cook University (Townsville, Queensland) and 15 viruses tested at the Australian Centre for Disease Preparedness (Geelong, Victoria).

## Results

**Fig. 1:**
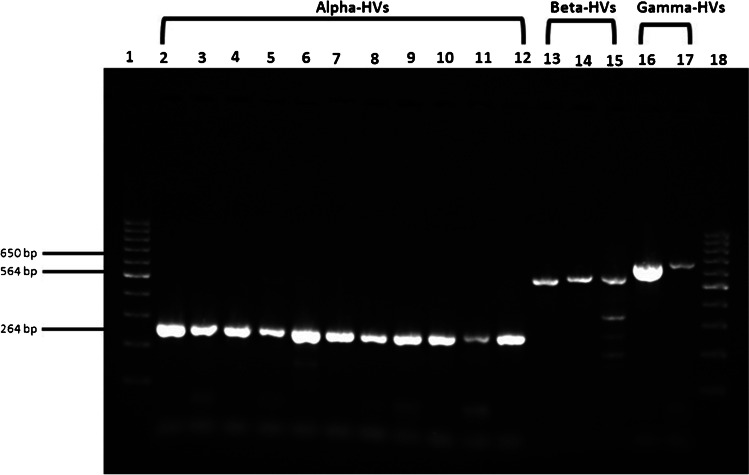
Electrophoresis of PCR products of HV DNAs obtained by STC-PCR in a 1.5% agarose gel. Lane 1 and 18 contain a 100 bp DNA marker; Lane 2 = *Bovine alphaherpesvirus 1*; lane 3 = *Chelonid alphaherpesvirus 5*, lane 4 = *Macropodid alphaherpesvirus 1*; lane 5 = *Macropodid alphaherpesvirus 2*; lane 6 = *Human alphaherpesvirus 1*; lane 7 = *Human alphaherpesvirus 2*; lane 8 = *Human alphaherpesvirus 3*; lane 9 = *Equid alphaherpesvirus 4*; lane 10 = *Meleagrid alphaherpesvirus 1*; lane 11 = *Gallid alphaherpesvirus 2*; lane 12 = *Felid alphaherpesvirus 1*; lane 13 = *Human betaherpesvirus 5*; lane 14 = *Human betaherpesvirus 6*; lane 15 = *Human betaherpesvirus 7*; lane 16 = *Human gammaherpesvirus 4*; lane 17 = *Human gammaherpesvirus 8*

Overall, a total of 56 primers targeting the conserved regions of different HV genes were designed and tested with a wide range of HV DNAs. Of these, the three primer pairs reported in this study (Table 1; Supplementary file 1, Figure S1) were found to sensitively amplify the DNA sequences of 32 HV species (Table 2). In addition, appropriately sized (specific) single bands were seen (for most of the HVs tested) on agarose gel following electrophoresis (Fig. 1). The addition of 5% DMSO and 15 mM TMAC greatly improved the sensitivity, specificity, and reproducibility of the PCR reaction (Supplementary file 1, Figure S2).

The detection limits (relative) of the STC-PCR were comparable or lower when compared to the previously reported nested-PCR (Table 2), except for human betaherpesvirus 6, for which the nested PCR detected the viral DNA at one 10-fold dilution lower (Supplementary file 1, Figure S3). The STC-PCR assays produced much ‘cleaner’ DNA gels than the nested-PCR, which consistently produced many non-specific bands.

Subfamily assay specificity tests showed that the primer pair of one subfamily did occasionally cross-amplify HV DNAs of the other subfamilies (Supplementary file 1, Figure S4). For instance, the alpha-HV primer pair (AlphaFWD1 and AlphaREV2) amplified the DNA of HHV-6 (faint band observed), a member of the subfamily *Betaherpesvirinae*, but did not amplify any gamma-HV DNA (Supplementary file 1, Figure S4). The beta-HV primer pair amplified the DNA of a gamma-HV, HHV-4 (faint band), but none of the alpha-HVs. The gamma-HV primers produced varying sized bands for some alpha-HVs including crocodyline HV1 (CrHV-1), meleagridid HV1 (MeHV-1), equine HV4 (EHV-4), bovine HV1 (BoHV-1), HHV-1 and HHV-2 (Supplementary file 1, Figure S4). None of the beta-HV DNA was amplified by the gamma-HV primers (Supplementary file 1, Figure S4).

## Discussion

Despite the biological and evolutionary divergence of HVs across the three subfamilies, many evolutionarily conserved core genes still persist [26, 27]. These genes encode proteins that play essential roles in viral entry, nucleic acid synthesis and metabolism, capsid maturation, and virion egress [26]. The DPOL and gB genes are among the most highly conserved genes of HVs and have previously been used as biomarkers for the detection of HVs [18, 19]. In this present study, a singleplex PCR assay targeting conserved genes (DPOL or gB genes) at the subfamily level was developed and successfully used to amplify a broad spectrum of human and animal HV DNAs. Also, the assay produced bright single bands on an electrophoretic gel, which is essential for downstream amplicon sequencing and identification of novel and known HVs.

The addition of 5% DMSO and 15 mM TMAC enhanced the STC-PCR by increasing product yield and ensuring assay reproducibility. High GC content is a common feature of HV genomes [28], and this could pose a challenge during amplification. As previously observed [29, 30], DMSO assists in reducing complex secondary structures and high melting temperature (Tm) associated with GC-rich templates, which in turn reduces duplex stability and allows efficient PCR. TMAC is often recommended when using degenerate primers and helps prevent mispriming by improving stringency of the PCR [31, 32].

In a previous study by VanDevanter et al. [19], a nested PCR using degenerate primers was found to have LODs ranging from a single copy to 100 copies of HV Polymerase DNA per 100 ng of human DNA. Therefore, the sensitivity of the STC-PCR relative to the nested PCR was determined using 10-fold dilutions of representative HVs. The assays were comparable or more sensitive than the nested assay across almost all of the herpesviruses tested. With the improved sensitivity, coupled with cost and time savings, the STC-PCRs can be employed for the epidemiological and clinical detection of known and novel HVs. Some cross-amplification between herpesvirus subfamilies was observed with the STC-PCR due to the high conservation of the targeted DPOL and gB genes at the family level. We consider this cross-amplification a universal feature of the STC-PCR for HV detection; therefore, positive results (amplicons) should be sequenced for onward identification and classification of the detected HVs.

Herpesviruses have been shown to be important pathogens across a large range of vertebrate hosts [1]. Recent initiatives to investigate viral diversity in wildlife hosts have utilised universal PCR assays to discover novel viruses, some with potential clinical and zoonotic concerns [33, 34]. For instance, universal PCR was used to identify six novel herpesviruses in multi-infected samples of chimpanzees (*Pan troglodytes verus*) [35]. Similarly, novel herpesviruses associated with respiratory disease in birds and hepatitis and enteritis in monitor lizards have been detected using universal PCR approaches [36, 37]. Although universal PCR assays have been an invaluable tool for these viral discovery initiatives, many of these assays can be problematic due to poor sensitivity, low specificity and contamination issues (especially with nested assays). Here, we have designed and evaluated novel singleplex universal PCR assays that will be useful for detection of known and novel herpesviruses from human and animal clinical samples.

## Supplementary information

ESM 1 (DOCX 7561 kb)

## Data Availability

All data analysed during this study are included in this published article [Supplementary file 1].
