# Supplementary material for: Development of subfamily-based consensus PCR assays for the detection of human and animal herpesviruses
Source: Eur J Clin Microbiol Infect Dis. 2023 Apr 21;42(6):741–6. doi: 10.1007/s10096-023-04605-w (PMC10172264; doi:10.1007/s10096-023-04605-w)
Supplement: Supplementary file 1 — (DOCX 7561 kb) [file 10096_2023_4605_MOESM1_ESM.docx]

**Table S1:** List of GenBank accession numbers for nucleotide sequences used for the design of consensus primers.

***Alphaherpesvirinae***

Varanid herpesvirus 2 (AB189433)

Varanid herpesvirus 1 (AY437559)

Tortoise alphaherpesvirus 4 (GQ222415)

Tortoise alphaherpesvirus 2 (AY916792)

Testudinid herpesvirus 3 (NC_027916)

Terrapene herpesvirus 1 (KJ004665)

Suid herpesvirus 1 (NC_006151)

Psittacid herpesvirus 1 (NC_005264)

Phocid alphaherpesvirus 1 (MH509440)

Passerid herpesvirus 1 (DQ287313)

Meleagrid herpesvirus 1 (NC_002641)

Macropodid herpesvirus 1 (NC_029132)

Macacine herpesvirus 1 (NC_004812.1)

Loggerhead orocutaneous herpesvirus (EU004542)

Loggerhead genital-respiratory herpesvirus (EU004539)

Lacerta viridis herpesvirus 1 (EU527336)

Iguanid herpesvirus 2 (AY236869)

Human herpesvirus 3 (KU529562.1)

Human herpesvirus 2 (AY038366.1)

Human herpesvirus 1 (MH697533.1)

Gerrhosaurid herpesvirus 3 (AF416630)

Gerrhosaurid herpesvirus 2 (AF416628)

Gerrhosaurid herpesvirus 1 (AF416629)

Gaviid herpesvirus 1 (GU130289)

Gallid herpesvirus 3 (NC_002577.1)

Gallid herpesvirus 2 (NC_002229.3)

Gallid alphaherpesvirus 1 (MF156851)

Equid herpesvirus 9 (NC_011644.1)

Equid herpesvirus 8 (NC_017826)

Equid herpesvirus 4 (KF434386.1)

Equid herpesvirus 3 (NC_024771)

Equid herpesvirus 1 (KF434381.1)

Emydoidea herpesvirus 1 (KY849379)

Emydid herpesvirus 1 (KF478668)

Duck enteritis virus (EF643560.1)

Columbid herpesvirus 1 (KJ995972.1)

Chelonid alphaherpesvirus 6 (EU006876)

Chelonid alphaherpesvirus 5 (NC_028891)

Bovine herpesvirus 5 (NC_005261.1)

Bovine herpesvirus 2 (MN086791.1)

Bovine herpesvirus 1.2 (KM258881.1)

***Betaherpesvirinae***

Macaca nemestrina herpesvirus 7 (NC_030200)

Porcine cytomegalovirus strain BJ09 (NC_022233)

Elephantid herpesvirus 1 (NC_020474)

Caviid herpesvirus 2 strain 21222 (NC_020231)

Murid herpesvirus 8 (NC_019559)

Saimiriine herpesvirus 4 strain SqSHV (NC_016448)

Aotine herpesvirus 1 strain S34E (NC_016447)

Cercopithecine herpesvirus 5 strain 2715 (NC_012783)

Human herpesvirus 5 strain Merlin (NC_006273)

Macacine herpesvirus 3 (NC_006150)

Tupaiid herpesvirus 1 (NC_002794)

Rat cytomegalovirus Maastricht (NC_002512)

Human herpesvirus 7 (NC_001716)

Human betaherpesvirus 6A (NC_001664)

Human herpesvirus 6B (NC_000898)

***Gammaherpesvirinae***

Suid gammaherpesvirus 4 (NC_038265)

Suid gammaherpesvirus 3 (NC_038264)

Saimiriine gammaherpesvirus 2 (NC_001350)

Ovine gammaherpesvirus 2 (NC_007646)

Murid gammaherpesvirus 4 (NC_001826)

Macacine herpesvirus 4 (NC_006146)

Macacine gammaherpesvirus 5 (NC_003401)

Human gammaherpesvirus 8 (NC_009333)

Human gammaherpesvirus 4 (NC_007605)

Felid gammaherpesvirus 1 (NC_028099)

Equid gammaherpesvirus 5 (NC_026421 - ORF9)

Equid gammaherpesvirus 2 (NC_001650)

Callitrichine gammaherpesvirus 3 (NC_004367)

Bovine gammaherpesvirus 6 (NC_024303)

Bovine gammaherpesvirus 4 (NC_002665)

Ateline gammaherpesvirus 3 (NC_001987)

Alcelaphine gammaherpesvirus 2 (NC_024382)

Alcelaphine gammaherpesvirus 1 (NC_002531)


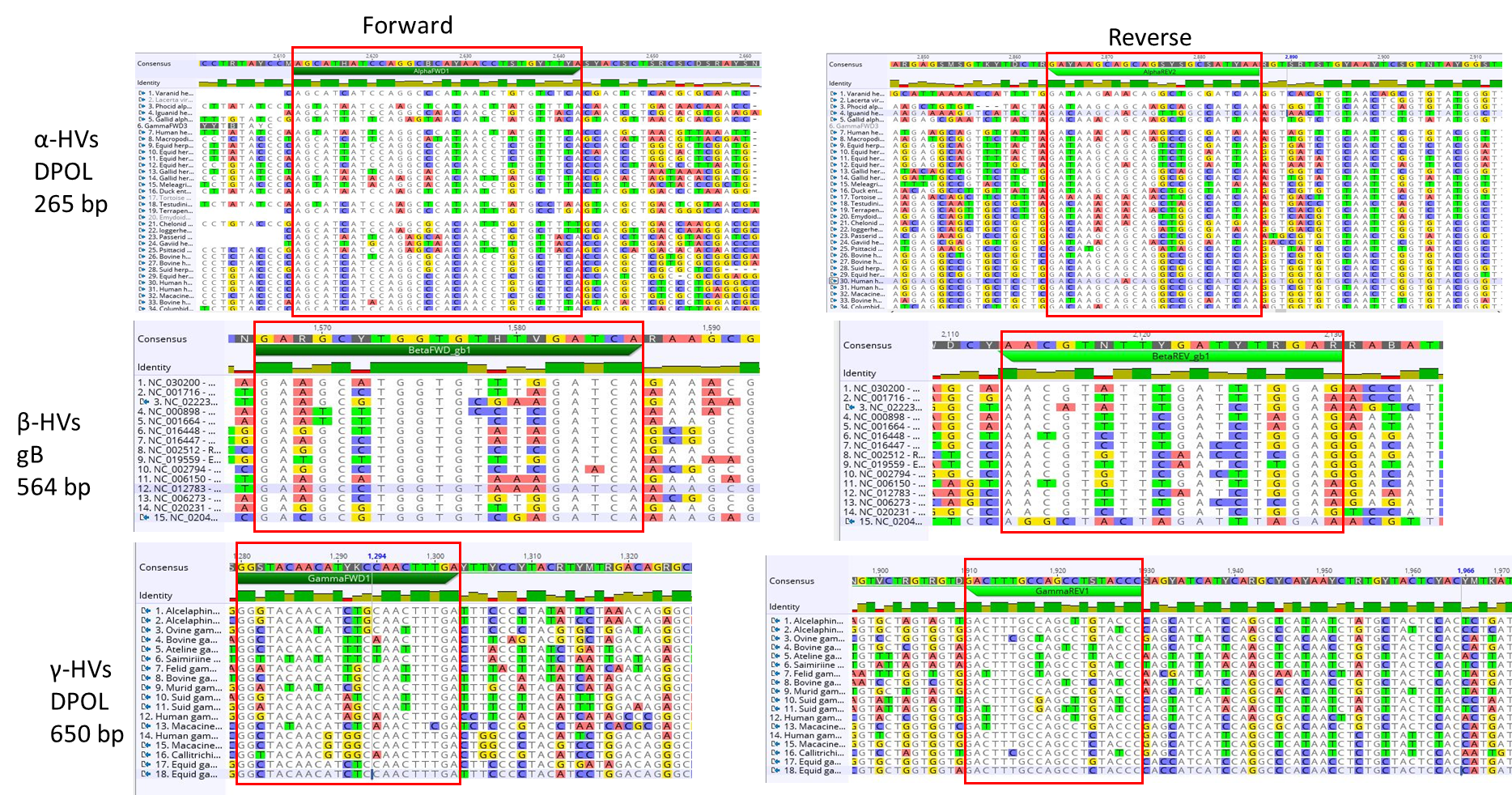


**Figure S1**: Multiple alignments for each of the viral subfamilies showing the primer binding sites. α-HVs= alphaherpesviruses; β-HVs= betaherpesviruses; γ-HVs= gammaherpesviruses; DPOL= DNA polymerase gene; gB= glycoprotein B gene.


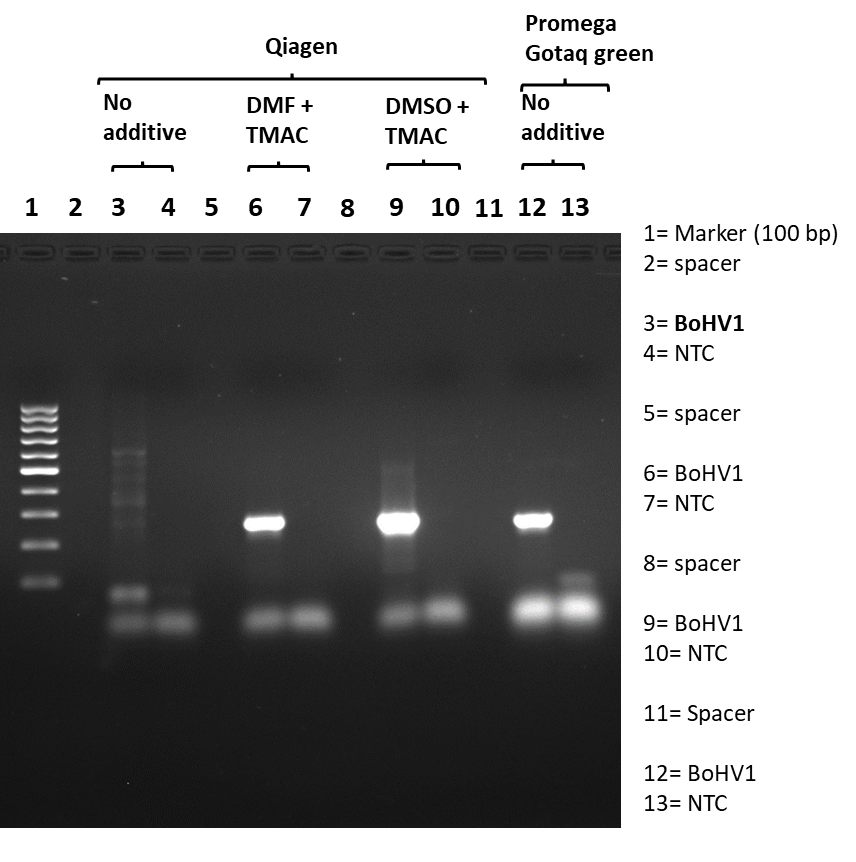


**Figure S2**: Effect of 2.5% DMF, 5% DMSO and 15mM TMAC with AlphaFWD1 and AlphaRev2 on the detection of *Bovine alphaherpesvirus 1* (BoHV1).


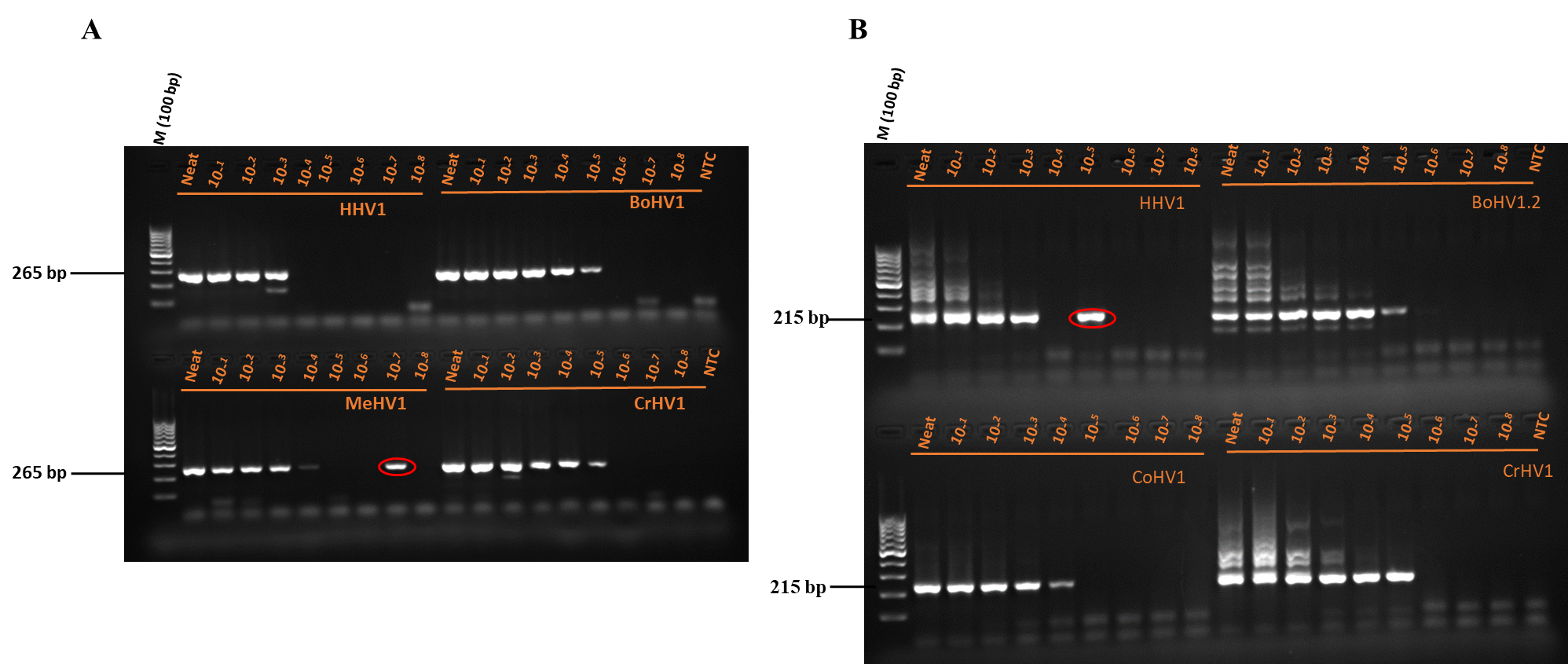


**
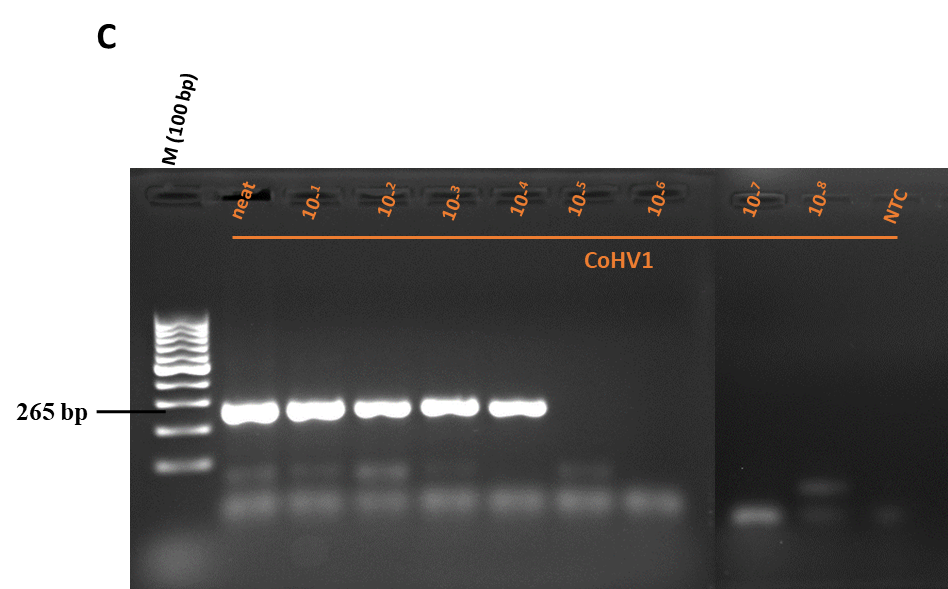
**

**
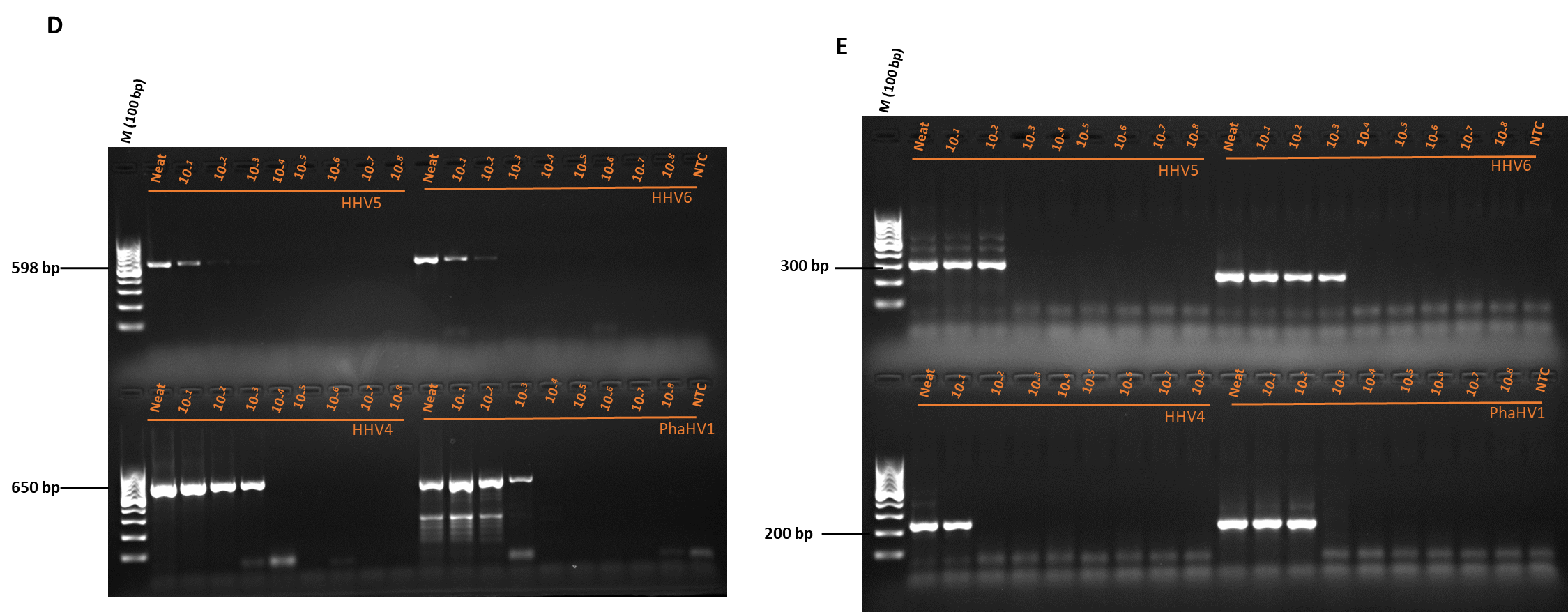
**

**Figure S3**: Comparison of the detection limit (relative sensitivity) of the singleplex touchdown PCR (A, C and D) and the nested PCR (B and E) for alphaherpesviruses (HHV1= *Human alphaherpesvirus 1*; BoHV1= *Bovine alphaherpesvirus 1*; MeHV1= *Meleagrid alphaherpesvirus 1* CrHV1= Crocodyline herpesvirus 1; CoHV1= *Columbid alphaherpesvirus*); betaherpesviruses (HH5= *Human alphaherpesvirus 5*; *Human alphaherpesvirus 6*); gammaherpesviruses (*Human alphaherpesvirus 4*; *Phascolarctid gammaherpesvirus 1*). Red cycled bands were excluded from this analysis as they were deemed to be contaminants.


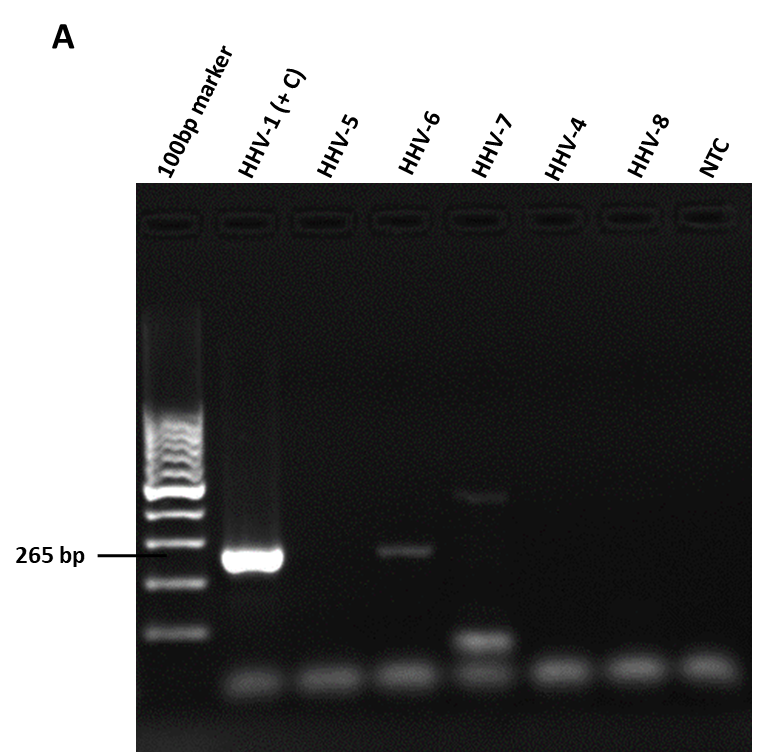


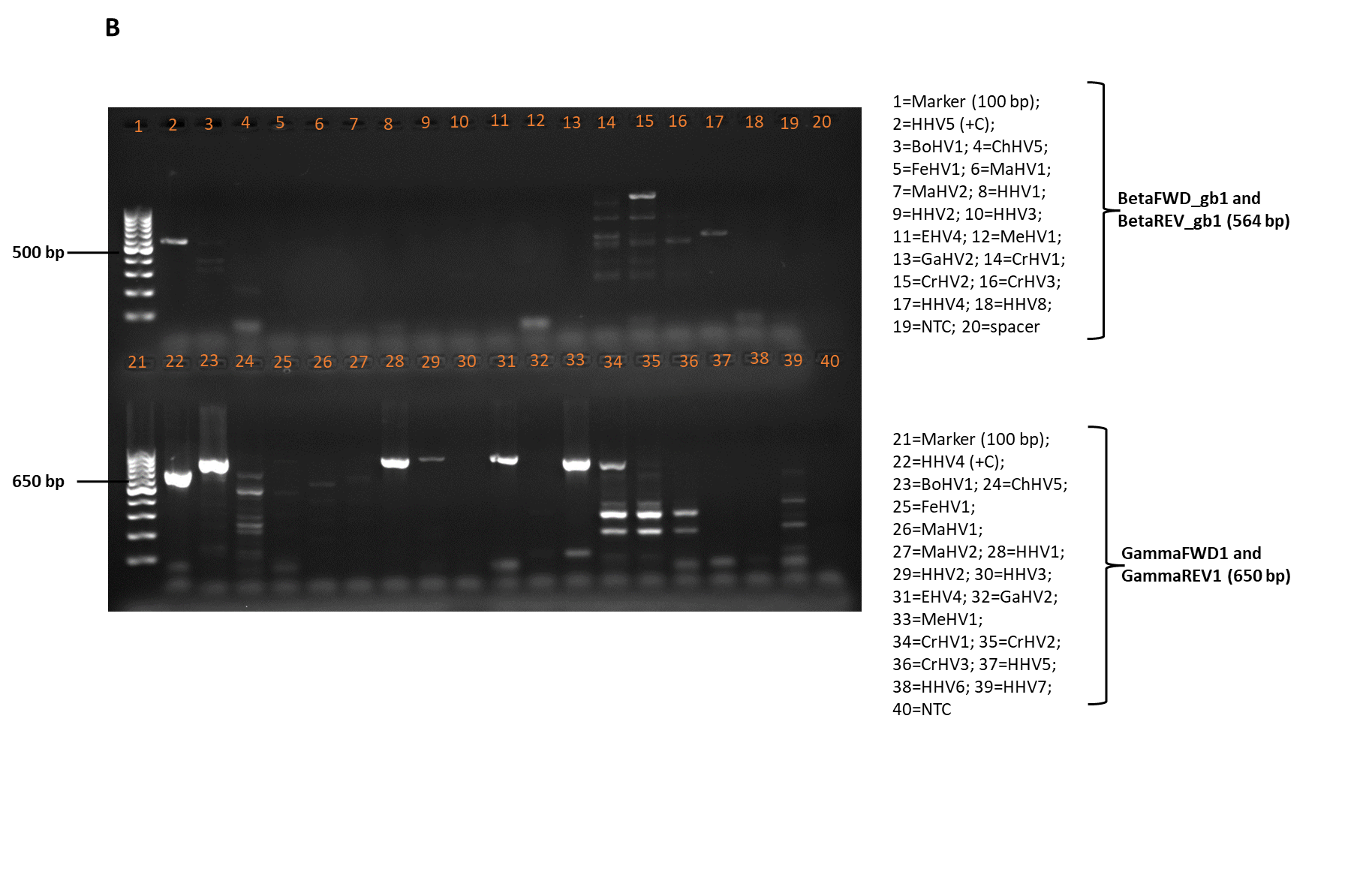


**Figure S4**: Specificity of the singleplex touchdown PCR (STC-PCR) for alphaherpesvirus (A; α-STC-PCR), betaherpesvirus (β-STC-PCR) and gammaherpesvirus (γ-STC-PCR) assays (B). The **α-STC-PCR** assay was used to test betaherpesviruses (HHV5=*Human alphaherpesvirus 5*; HHV6=*Human alphaherpesvirus 6;* HHV7=*Human alphaherpesvirus 7*) and gammaherpesviruses (HHV4=*Human alphaherpesvirus 4*; HHV8=*Human alphaherpesvirus 8*). The **β-STC-PCR** assay was used to test alphaherpesviruses (BoHV1= *Bovine alphaherpesvirus 1*; ChHV5=*Chelonid alphaherpesvirus 5*; FeHV1=*Felid alphaherpesvirus 1*; MaHV1=*Macropodid alphaherpesvirus 1*; MaHV2=*Macropodid alphaherpesvirus 2*; HHV1=*Human alphaherpesvirus 1*; HHV2=*Human alphaherpesvirus 2*; HHV3=*Human alphaherpesvirus 3;* EHV4=*Equid alphaherpesvirus 4*; MeHV1=*Meleagrid alphaherpesvirus 1*; GaHV2=*Gallid* *alphaherpesvirus 2*; CrHV1=Crocodyline herpesvirus 1; CrHV2=Crocodyline herpesvirus 2; CrHV3=Crocodyline herpesvirus 3) and gammaherpesviruses (HHV4 and HHV8). The **γ-STC-PCR** assay was used to test alphaherpesviruses (BoHV1; ChHV5; FeHV1; MaHV1; MaHV2; HHV1; HHV2; HHV3*;* EHV4; MeHV1; GaHV2; CrHV1; CrHV2; CrHV3) and betaherpesviruses (HHV5; HHV6; HHV7). Positive control (+C) and no template control (NTC) were included in all of the experiments.
